# Bioceramic Surface Topography Regulating Immune Osteogenesis

**DOI:** 10.34133/bmef.0089

**Published:** 2025-01-23

**Authors:** Jianxin Hao, Lin Du, Yuening He, Chengtie Wu

**Affiliations:** ^1^ State Key Laboratory of High-Performance Ceramics and Superfine Microstructure, Shanghai Institute of Ceramics, Chinese Academy of Sciences, Shanghai 200050, P. R. China.; ^2^ Center of Materials Science and Optoelectronics Engineering, University of Chinese Academy of Sciences, Beijing 100049, P. R. China.

## Abstract

**Objective:** This study aims to clarify the effects of bioceramic interface cues on macrophages. **Impact Statement:** Recently, there have been many researches exploring the effects of interface topography cues on macrophage polarization and cytokine secretion. However, the effects and underlying mechanisms of bioceramic interface cues on macrophages still need exploring. This study provides insights into the effects of bioceramic micro-groove surface structures on macrophages. **Introduction:** With the development of bone tissue engineering methods, bioceramics have been used for bone repair. After the implantation of bioceramics, innate immune response that occurs at the interface of materials can deeply influence the subsequent inflammation and bone regeneration progress. Therefore, the exploration and regulation of immune response of the bioceramic interface will be beneficial to promote the bone regeneration effects. **Methods:** In this study, bioceramics with micro-groove structures on the surface are fabricated by digital light processing 3-dimensional printing technology. Then, micro-groove structures with different spacings (0, 25, 50, and 75 μm) are prepared separately to explore the effects on macrophages. **Results:** The large spacing micro-groove structure can promote the M2 polarization and osteoinductive cytokine secretion of macrophage. The reason is that the large spacing micro-groove structure can induce directional arrangement of macrophage so as to change the phenotype and cytokine secretion. Further researches show that macrophage of the large spacing micro-groove structure can promote the osteogenic differentiation of bone mesenchymal stem cells, which can benefit osteogenesis and osteointegration. **Conclusion:** This study offers an effective and application potential method for bone repair.

## Introduction

With the development of bone tissue engineering, tissue engineering scaffolds, especially of bioceramics, have become the major and most application potential method for bone regeneration [1–4]. Because of the bone-like mechanical properties and well osteoconductive ability, bioceramics could provide mechanical support and induce the formation of new bone for bone defect [5,6]. Moreover, ions released from bioceramics could also regulate multiple cell interactions and activate different signaling pathways to promote the process of bone regeneration [7]. However, although the bone regeneration effects of bioceramics have been widely studied and clarified, the influence on bone immune response still needs to be explored. After the implantation of bioceramics, macrophages would first arrive at the site of implantation [8,9] and mediate the immunoreaction at the interface of bioceramics [10]. Because of high cell plasticity, macrophages could mediate various immune responses and influenced the functions of bone marrow mesenchymal stem cells (BMSCs) by polarizing as M1 or M2 phenotype [11]. M1 phenotype was the main type of macrophages at the inflammation site for initial few hours, which could mediate the early immune inflammation by secreting various inflammatory factors, such as tumor necrosis factor-α (TNF-α) [12]. Moreover, M1 phenotype macrophages could also recruit BMSCs into the implantation sites. However, these immune responses mediated by M1 macrophages could only benefit the early osteogenesis stage. Persistently, abundant and long-term existence of M1 macrophages at the implantation sites could lead to the formation of fibrotic scar tissue and bone regeneration delay [13]. Interestingly, M2 macrophages could promote the osteogenic differentiation by secreting various anti-inflammatory factors, such as interleukin-10 (IL-10) and transforming growth factor-β (TGF-β) [14]. The transformation from M1 to M2 was the key stage of bone immune response, which also influenced the effects of osteogenesis and osteointegration of bioceramics. Therefore, the exploration and regulation of bone immune response of bioceramics would be beneficial to promote the bone regeneration effects.

Over the past few years, researchers have tried to regulate the immune microenvironment of bioceramics and promote immune osteogenesis via various biochemical cues (such as ion release and mineralization) [11,15]. Recent evidences suggested that, because bone immune response usually occurred at the interface of bioceramics, surface topographical cues played important roles in the regulation of immune microenvironment and subsequent bone repair. Many conducted researches have shown that different surface topographical cues could influence the function, secretion, and phenotype of macrophages by regulating the cell morphology, cell arrangement, or cell behavior [16–21]. However, most of the previous researches on surface topographical cues often used titanium as materials, which could not fully represent the results of bioactive materials. For deeply understanding the interactions between bioceramics and macrophages, regulating the immune microenvironment, and further promoting the effects of immune osteogenesis, the influences and mechanisms of bioceramic interface topographical cues on macrophages still needed exploration.

**Fig. 1.**
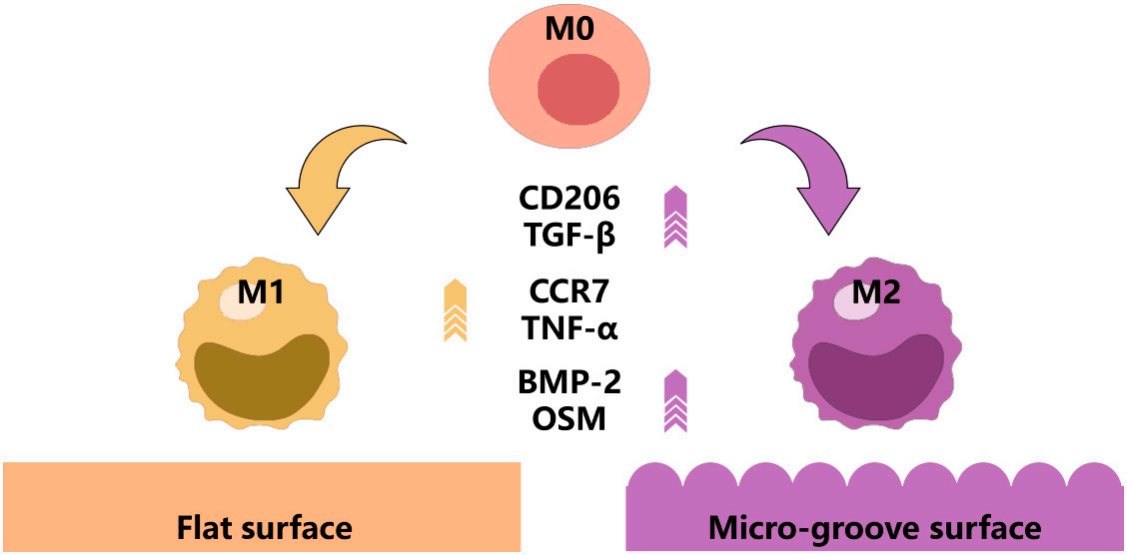
Scheme of the effects of bioceramics with flat surface and different spaced micro-groove structures on arrangement, cytokine secretion, and phenotype transformation of macrophages.

In this study, β-tricalcium phosphate (β-TCP) bioceramics with micro-groove structures on the surface were fabricated by digital light processing (DLP) 3-dimensional (3D) printing technology. Then, to evaluate the effects on cell arrangement, cytokine secretion, and phenotype transformation of macrophages, bioceramics with flat surface and different spaced micro-groove structures were designed and prepared for macrophage culture (Fig. 1). Moreover, the osteogenic ability of BMSCs was further evaluated by the conditioned medium (CM) of macrophages growing on bioceramics with different spaced micro-groove structures.

## Results

### Fabrication and characterization of bioceramics with micro-groove structures on the surface

**Fig. 2.**
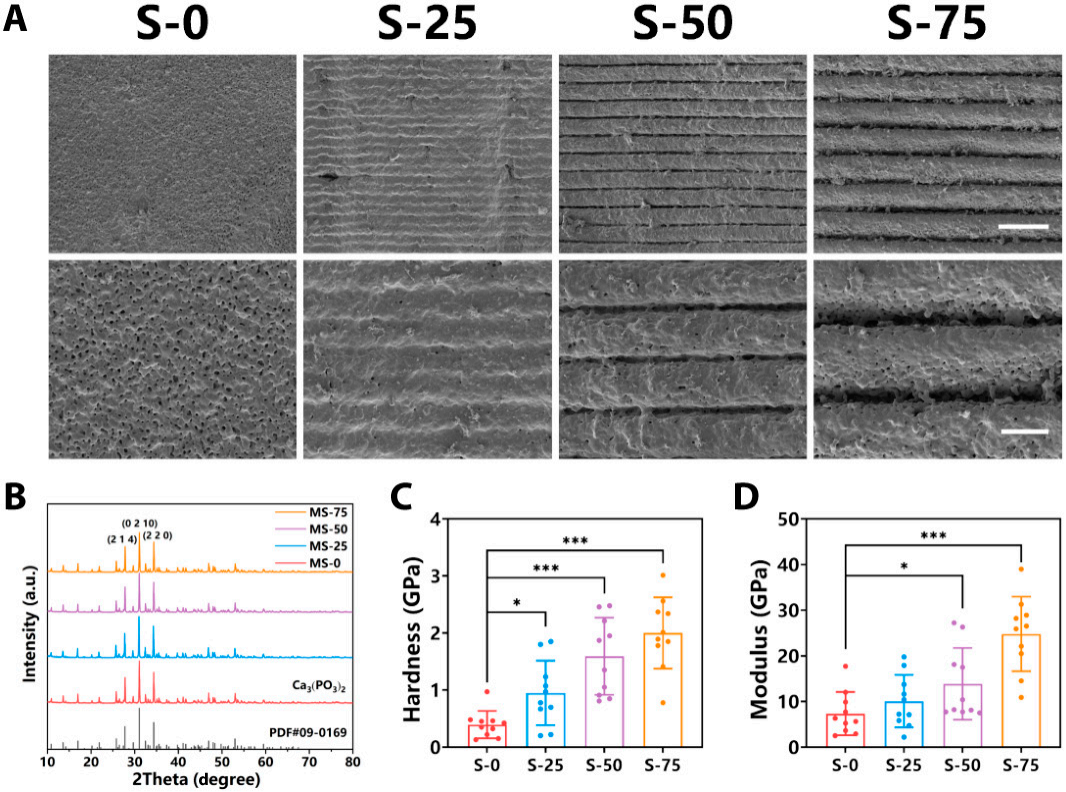
The morphology and mechanical properties of bioceramics with different spaced micro-groove structures on the surface. (A) SEM images of bioceramics with different spaced micro-groove structures on the surface. Scale bars, 100 μm (top) and 30 μm (bottom). (B) XRD of bioceramics with different spaced micro-groove structures on the surface. (C) Hardness of bioceramics with different spaced micro-groove structures on the surface. (D) Modulus of bioceramics with different spaced micro-groove structures on the surface. Data are means ± SD. *n* = 10. *P < 0.05, **P < 0.01, ***P < 0.001. The bioceramics with different spaced micro-groove structures on the surface were intact and well sintering.

To fabricate β-TCP bioceramics with micro-groove structures on the surface, the layer-by-layer forming method of DLP 3D printing technology was used. By regulating the thickness of curing layer, micro-groove structures with different spacings could be fabricated on the surface of bioceramics. Considering the size of RAW 264.7 (10 to 30 μm), the spacings of micro-groove were designed as 0, 25, 50, and 75 μm in order to effectively induce the arrangement and phenotype transformation of macrophages. Figure 2A shows bioceramics with micro-groove structures of 4 spacings (0, 25, 50, and 75 μm, named as S-0, S-25, S-50, and S-75, respectively). S-0 was bioceramic with flat surface, while S-25, S-50, and S-75 were bioceramics with different spaced micro-groove structures on the surface. High-magnification scanning electron microscope (SEM) images of bioceramic surfaces showed that all of the bioceramics were well sintered, which was the foundation of mechanical properties and cell adhesion. The surface of S-25 exhibited a gentle fluctuation, while the surface of S-50 and S-75 showed obvious micro-grooves. Moreover, SEM images of sections showed the surface profile of bioceramics more clearly. The micro-grooves of S-25, S-50, and S-75 were semicircular, and the depth of micro-grooves was 10, 25, and 35 μm, respectively (Fig. S1). The results of x-ray diffraction (XRD) showed that micro-groove structures were pure β-TCP bioceramic phase, and the organic phase had been removed (Fig. 2B). The complete removal of organic phase promised that micro-groove structures had better biocompatibility and degradability. Computed tomography was used to measure the porosity of different spacing bioceramics. Results showed that S-0, S-25, S-50, and S-75 have similar porosity, which were 47.01%, 47.20%, 49.92%, and 47.01%, respectively (Fig. S2). Moreover, nano-indentation assay was used to measure the mechanical properties of bioceramic surfaces with different spaced micro-groove structures. As shown in Fig. 2C and D, the mean hardness and modulus of S-0 were 0.55 and 10.1 GPa, respectively, while those of S-25, S-50, and S-75 were 1.025 and 11 GPa, 1.645 and 17.35 GPa, and 1.895 and 24.95 GPa, respectively. The increase of mean hardness and modulus with micro-groove spacing indicated that different spaced micro-groove structures could promote the mechanical properties of bioceramic surfaces. The reason for this phenomenon was because the increased thickness of curing layer could promote the quality of sintering. As the results of SEM, the surface of S-0 had uniform pore, while S-25, S-50, and S-75 had fewer micropores and denser surfaces. Moreover, the surface roughness of S-0, S-25, S-50, and S-75 was (48.8 ± 7.4) μm, (52.4 ± 5.0) μm, (38.3 ± 2.6) μm, and (41.3 ± 1.2) μm, respectively (Table S1). The change of surface roughness also confirmed the effects of curing layer thickness on the quality of sintering.

### Directional arrangement and phenotype transformation of macrophages

**Fig. 3.**
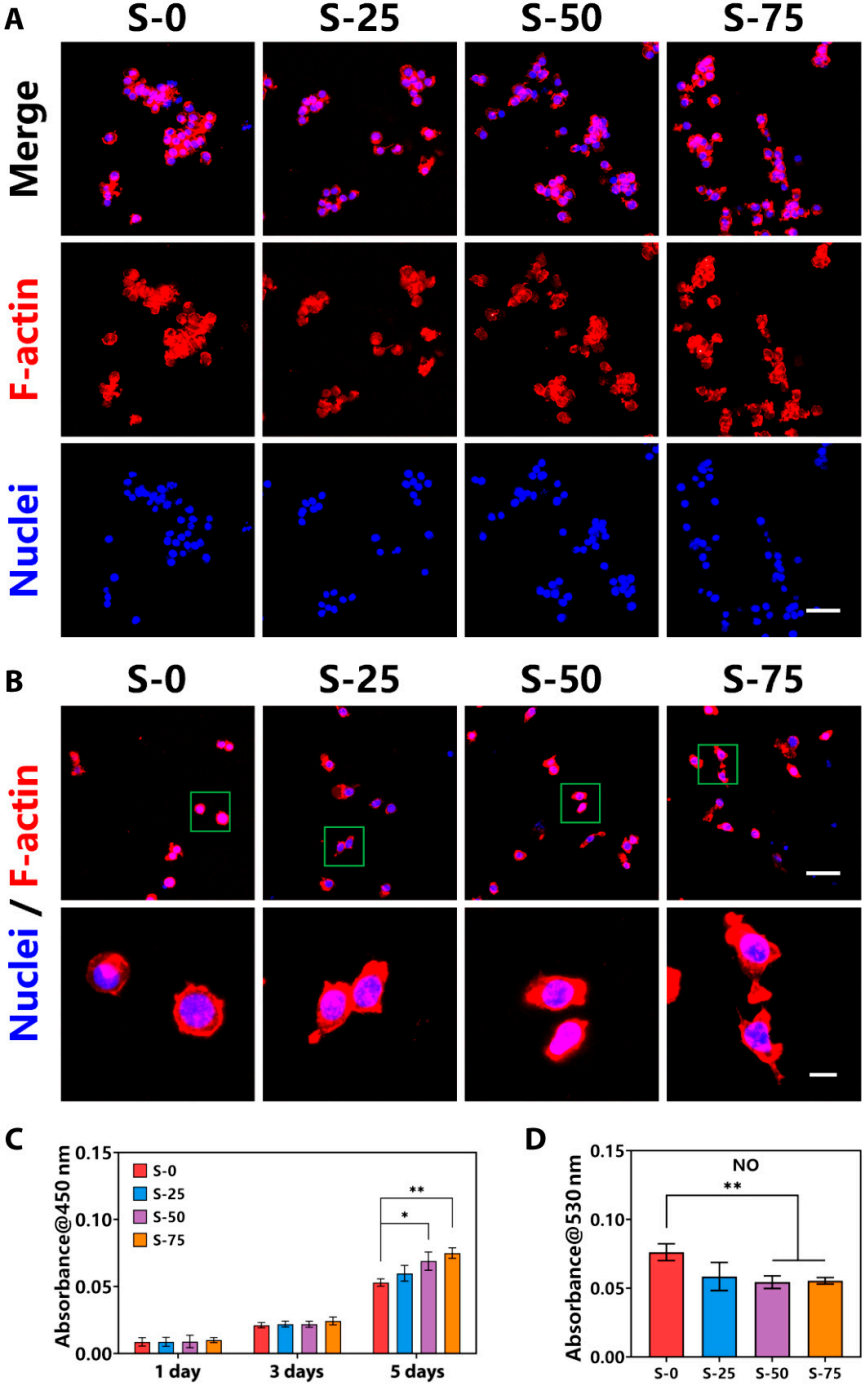
The cell arrangement, proliferation, and morphology of macrophages that adhere on bioceramic surfaces with different spaced micro-groove structures. (A) CLSM images of macrophage arrangement of different groups (red: F-actin, 488 nm; blue: nuclei, 405 nm; scale bar, 50 μm). (B) CLSM images of macrophage morphology of different groups [red: F-actin, 488 nm; blue: nuclei, 405 nm; scale bars, 50 μm (top) and 10 μm (bottom)]. (C) Proliferation activity of macrophages for 5 d. (D) NO secreting activity of macrophages for 5 d. Data are means ± SD. *n* = 3. *P < 0.05, **P < 0.01, ***P < 0.001. Macrophages adhering on the bioceramic with the 75 μm spaced micro-groove structure had directional tendency of cell arrangement and higher cell growth rate.

After successfully obtaining bioceramics with different spaced micro-groove structures on the surface, macrophages were seeded on bioceramic surfaces to evaluate the effects of micro-groove structures. Confocal laser scanning microscopy (CLSM) images showed that macrophages distributed on the surface of S-0 randomly and grew in clusters, similar to those of S-25. Interestingly, macrophages adhering on the surface of S-50 and S-75 exhibited a directional tendency of arrangement and grew along the micro-grooves, especially S-75 (Fig. 3A). Although exhibiting a directional tendency in whole, macrophages also grew in clusters locally, which were in accordance with the natural growing stage. In addition, high-magnification CLSM images indicated that the morphology of most macrophages of S-0 was spherical shapes, while the morphology of some macrophages on S-75 exhibited spindle shapes (Fig. 3B). Similarly, the morphology of S-25 and S-50 also exhibited approximately spindle shapes. The directional tendency of arrangement and spindle shapes of cell morphology seemed to be beneficial for the adhesion and proliferation of macrophages (Fig. 3C). Moreover, previous researches suggested that morphology transformation of macrophages may be related to the change of function and secretion, and also represented different phenotypes. Generally, macrophages with circle morphology was often considered as the typical M0 phenotype, while macrophages with multi-pseudopodia and larger cell volume usually represented the pro-inflammatory M1 phenotype, and spindle shape represented the anti-inflammatory M2 phenotype [22]. These evidences suggested that bioceramic with a suitable spaced micro-groove structure on the surface may be able to promote the directional cell arrangement, growth rate, and M2 phenotype transformation of macrophages.

**Fig. 4.**
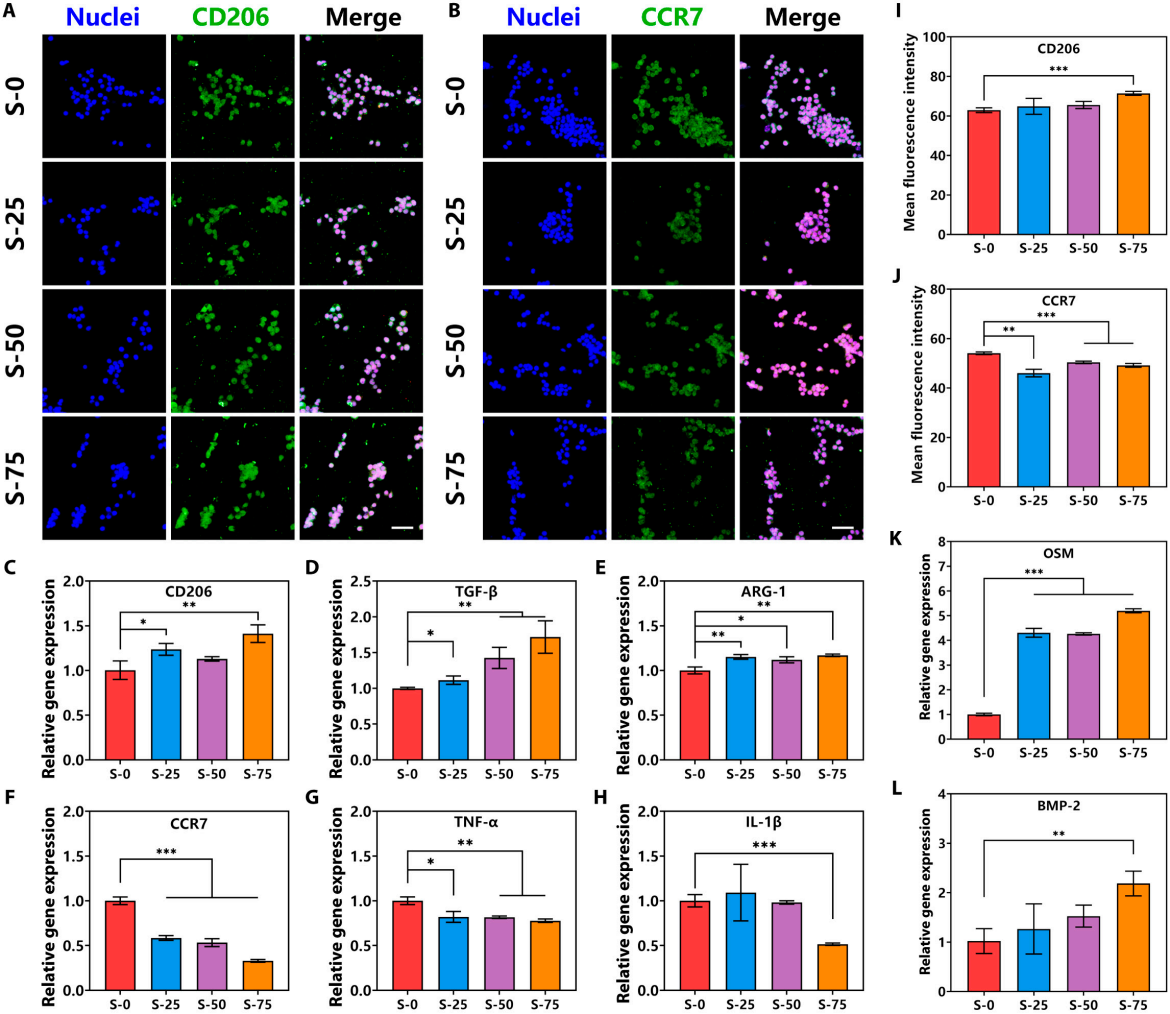
The expression of phenotype-related genes and proteins of macrophages after culturing on the bioceramic surface with different spaced micro-groove structures. (A) Immunofluorescence staining images of M2 phenotype marker CD206 of different groups (green: CD206, 647 nm; blue: nuclei, 405 nm; scale bar, 50 μm). (B) Immunofluorescence staining images of M1 phenotype marker CCR7 of different groups (green: CCR7, 647 nm; blue: nuclei, 405 nm; scale bar, 50 μm). (C to E) Expression of M2 phenotype-related genes CD206, TGF-β, and ARG-1 of different groups for 5 d. (F to H) Expression activity of M1 phenotype-related genes CCR7, TNF-α, and IL-1β of different groups for 5 d. (I) Semiquantitative analysis of CD206 of different groups. (J) Semiquantitative analysis of CCR7 of different groups. (K) Expression of OSM of different groups. (L) Expression of BMP-2 of different groups. Data are means ± SD. *n* = 3. *P < 0.05, **P < 0.01, ***P < 0.001. Macrophages adhering on the bioceramics with 75 μm spaced micro-groove structures had higher proportion of M2 phenotype and expression of osteoinductive cytokines.

To further evaluate the effects of different spaced micro-groove structures on the phenotype transformation of macrophages, the typical secreting cytokines and markers of macrophage phenotype were measured. Nitrogen monoxide (NO), related to the activation of inducible nitric oxide (NO) synthase and metabolism of arginine, was the main metabolite of M1 phenotype macrophages, while M2 phenotype macrophages were hardly secreted [23]. Thus, the measurement of NO secreting activity was quite necessary for the evaluation of macrophage phenotype. Figure 3D shows that macrophages adhering on S-0 had the highest NO concentration after culturing for 5 d, which meant the highest cell proportion of M1 phenotype macrophages. At the same time, macrophages growing on S-75 showed obviously decreased concentration of NO, meaning lower cell proportion of M1 phenotype macrophages. Immunofluorescent staining was also used to evaluate the phenotype transformation of macrophages on different spaced micro-groove structures. CC chemokine receptor 7 (CCR7) and mannose receptor 1 (MRC1; also named as CD206) were the typical membrane protein markers of M1 and M2 phenotype macrophages, respectively [23–26]. Figure 4A and I shows the immunofluorescence staining images and results of semiquantitative fluorescence intensity of CD206. Figure 4B and J shows the images and results of CCR7. Compared with the randomly arranged macrophages on S-0, directionally arranged macrophages on S-75 had higher expression activity of CD206 and lower expression activity of CCR7. Moreover, further studies on phenotype-related gene expression showed that macrophages adhering on bioceramic surfaces with different spaced micro-groove structures had higher expression activity of M2 phenotype-related genes CD206, TGF-β, and ARG-1 (Fig. 4C to E) and lower expression activity of M1 phenotype-related genes CCR7, TNF-α, and IL-1β (Fig. 4F to H). These effects were particularly noticeable on macrophages of S-75, which means that the bioceramic surface with the 75 μm spaced micro-groove structure could promote the phenotype transformation of macrophages into M2 phenotype. The above results showed that the bioceramic with the 75 μm spaced micro-groove structure on the surface could help the directional arrangement of macrophages, while macrophages on the flat surface and 25 μm spaced micro-groove structure surface were random arrangement. Interestingly, the transformation from random cell arrangement into directional cell arrangement was able to further influence the functions of macrophages. Specifically, directionally arranged macrophages would have higher proliferation activity and expression of M2 phenotype-related genes CD206, TGF-β, and ARG-1.

### Osteoinductive ability of macrophages on BMSCs

Numerous researches had demonstrated that the M2 phenotype macrophages could benefit osteogenesis and osteointegration, because of the regulation of inflammatory environment, and promoted secretion of osteoinductive cytokines [27]. As mentioned above, bioceramics with the 75 μm spaced micro-groove structure on the surface could help the cell rearrangement of macrophages and then could promote the phenotype transformation to M2. The increased M2 phenotype proportion of macrophages could influence the secretion of osteoinductive cytokines. Results of osteoinductive-related gene expression showed that macrophages on S-75 had higher expression of BMP-2 and OSM (Fig. 4K and L), which were important factors of bone growth and could promote the subsequent bone regeneration [28].

**Fig. 5.**
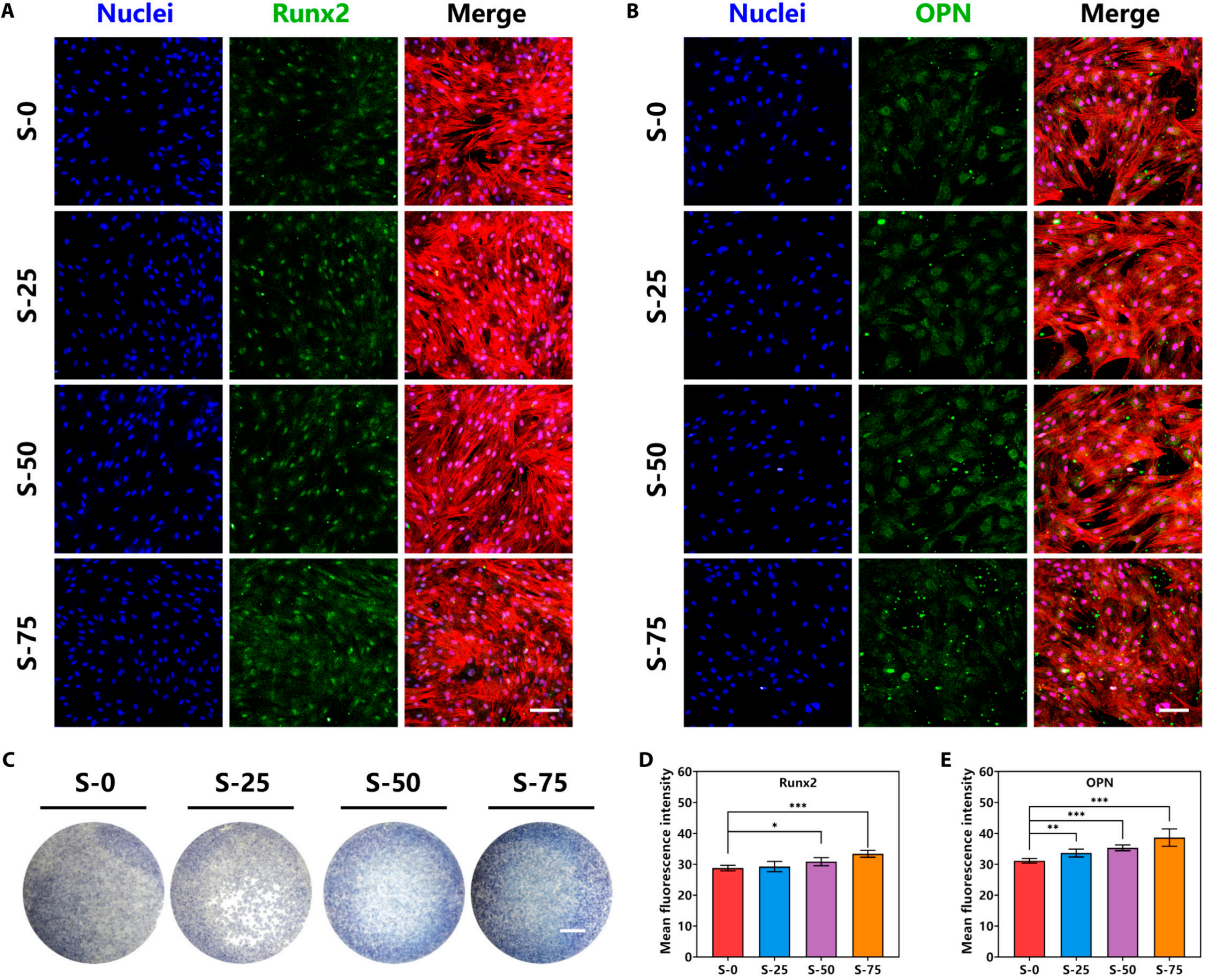
The osteogenic activity of BMSCs cultured with CM of macrophages of S-0, S-25, S-50, and S-75. (A) Immunofluorescence staining images of early osteogenic signature protein Runx2 of different groups for 5 d (green: Runx2, 647 nm; blue: nuclei, 405 nm; scale bar, 100 μm). (B) Immunofluorescence staining images of late osteogenic signature protein OPN of different groups for 7 d (green: OPN, 647 nm; blue: nuclei, 405 nm; scale bar, 100 μm). (C) ALP staining images of different groups for 7 d (scale bar, 1 mm). (D) Semiquantitative analysis of Runx2 of different groups. (E) Semiquantitative analysis of OPN of different groups. Data are means ± SD. *n* = 5. *P < 0.05, **P < 0.01, ***P < 0.001. CM of macrophages adhering on bioceramic with the 75 μm spaced micro-groove structure could promote the osteogenic differentiation of BMSCs.

To clarify the effects on the osteogenic differentiation of BMSCs, CM of macrophages adhering on bioceramics with different spaced micro-groove structures was collected to culture BMSCs. After culturing for 7 d, immunofluorescent staining assay was used to evaluate the osteogenic differentiation level of BMSCs. Runx2 was a key transcriptional factor of differentiation progress from BMSCs into osteoblasts, influencing many signaling pathways of osteogenic differentiation [29]. Moreover, ALP and OPN were important marker proteins of early and late stage of osteogenic differentiation separately [30,31]. Therefore, the expression of Runx2 and OPN was measured by immunofluorescent staining assay. Figure 5A and B shows the results of immunofluorescent staining assay. Compared with the BMSCs cultured with macrophage CM of S-0, BMSCs cultured with macrophage CM of different spaced micro-groove structures had higher protein expression of Runx2 (Fig. 5D) and OPN (Fig. 5E). The expression activity increased as the spacing of micro-groove, and macrophage CM of S-75 had best promoting effects of the expression of Runx2 and OPN. In addition, ALP staining assay was used to observe ALP expression of BMSCs (Fig. 5C). Similarly, ALP staining images of BMSCs cultured with macrophage CM of S-75 had deeper blue-violet, meaning obviously increased expression of ALP. The above results showed that directionally arranged macrophages, adhering on bioceramics with 75 μm spaced micro-groove structures, could secrete more osteoinductive cytokines and then promote the osteogenic differentiation and bone regeneration effects of BMSCs. Therefore, suitable immune microenvironment provided by bioceramic with the 75 μm spaced micro-groove structure could promote better osteogenesis and osteointegration.

## Conclusion

This study successfully designed and fabricated bioceramics with different spaced micro-groove structures to evaluate the roles of bioceramic topography cues on macrophages. By culturing macrophages on 0, 25, 50, and 75 μm spaced micro-groove structures separately, the effects and mechanisms on macrophage arrangement, cell proliferation activity, phenotype transformation, and cytokine secretion were elucidated. The osteoinductive ability of macrophages on BMSCs was further evaluated by CM method. Results showed that bioceramics with large spaced micro-groove structures on the surface could induce the directional arrangement of macrophages. Compared with the randomly arranged cells on the flat surface and small spaced micro-groove structures, the directionally arranged macrophages often had higher proliferation activity and larger proportion of M2 polarization phenotype. Moreover, the directionally arranged macrophages also secreted more osteoinductive factors to promote the osteogenic differentiation of BMSCs. Overall, this study provided a practical and application potential method to promote the immune osteogenic effects of bioceramics.

## Materials and Methods

### Materials

β-TCP was brought from Kunshan Chinese Technology New Materials Co. Ltd. (China). Photosensitive resin was brought from eSUN 3D Printing Co. Ltd. (China).

### Fabrication of bioceramic with surface microstructure

The green body of bioceramic with the surface microstructure was fabricated by bioceramic slurry with a DLP 3D printer (AUTOCERA-M, Beijing Ten Dimensions Technology Co. Ltd., China). The bioceramic slurry consisted of 45 g of β-TCP powder and 55 g of photosensitive resin, which had been ball milled for 3 h. Then, the bioceramic slurry was poured into the 3D printer and cured under ultraviolet light slice by slice. To establish the surface microstructure of bioceramic, the slurry was cured specifically with the slice thickness of 25, 50, and 75 μm. Finally, all of the green bodies were sintered at 1,150 °C to obtain bioceramic with the surface microstructure.

### Characterization of bioceramic with surface microstructure

The morphology of the microstructure of surface and section was observed by SEM (SU8220, Hitachi, Japan). The material phase of bioceramic was observed by XRD (Ultima IV, Rigaku, Japan). The porosity of bioceramic was measured by computed tomography (Skyscan1172, Bruker, Germany). The surface roughness was measured with a surface profilometer (Daktak-XT, Bruker, USA).

### Cell isolation and culture

RAW 264.7 was supplied by Cell Bank of the Chinese Academy of Sciences (Shanghai, China) and subcultured in Dulbecco’s modified Eagle’s medium (DMEM; Biological Industries, Israel) added with 1% penicillin/streptomycin (Invitrogen, USA) and 10% fetal bovine serum (FBS; Invitrogen, USA).

BMSCs were isolated by the femur of rabbits and subcultured in α-minimum essential medium (α-MEM; Gibco, USA) added with 1% penicillin/streptomycin (Invitrogen, USA) and 10% FBS (Invitrogen, USA), as reported previously [32].

### Cell adhesion and proliferation assay

RAW 264.7 was seeded on the surface of bioceramic slice and cultured in DMEM for 5 d. Unless otherwise specified, this method would be used in subsequent experiments.

For cell adhesion assay, bioceramic slices were fixed with 4% paraformaldehyde (PFA) in phosphate-buffered saline (PBS) and then stained by 4′,6-diamidino-2-phenylindole (DAPI; nuclei, excitation = 405 nm) and fluorescein isothiocyanate-labeled phalloidin (FITC; F-actin, excitation = 488 nm). Fluorescence images of cell morphology were observed by CLSM (TCS SP8, Leica, Germany).

For cell proliferation assay, cell counting kit-8 assay (CCK-8; Beyotime, China) was used to evaluate the proliferation rate of cell.

### Macrophage phenotype assay

NO assay was used to assess the proportion of M1 macrophage. RAW 264.7 seeded on the surface of bioceramic slices was collected and lysed into protein sample by cell lysis buffer for Western blotting and immunoprecipitation (Beyotime, China). Then, the NO concentration of protein sample was measured by Nitric Oxide Assay Kit (Beyotime, China).

Immunofluorescence staining was used to measure the expression of macrophage phenotype markers. RAW 264.7 seeded on the surface of bioceramic slices with different surface microstructures was fixed by 4% PFA, permeabilized by 0.5% Triton X-100, and blocked by 1% bovine serum albumin (BSA). Then, bioceramic slices were treated with primary antibody, fluorescent secondary antibody (CD206 and CCR7, excitation = 647 nm), FITC, and DAPI in turn. Protein fluorescence images were observed by CLSM (TCS SP8, Leica, Germany). The number of parallel samples was 3.

### BMSC osteogenic differentiation assay

BMSCs were seeded in 12-well cell culture plates with the density of 50,000 cells per hole and cultured in CM of macrophage. Specifically, RAW 264.7 was seeded on the surface of bioceramic slices with different surface microstructures and cultured in DMEM for 3 d. Then, the culture medium was collected and mixed with an equal amount α-MEM to obtain CM.

Alkaline phosphatase (ALP) and Alizarin Red S (ARS) staining were used to measure the osteogenic differentiation ability of BMSCs. For ALP staining assay, BMSCs seeded in 12-well cell culture plates were fixed with 4% PFA, and then ALP was stained by BCIP/NBT Alkaline Phosphatase Color Development Kit (Beyotime, China). For ARS staining assay, BMSCs were also fixed with 4% PFA, and then ARS was stained by Alizarin Red S Staining Kit for Osteogenesis (Beyotime, China).

Immunofluorescence staining was used to measure the expression of osteogenic proteins. BMSCs were seeded on the surface of cell slides and cultured in CM for 5 d. Cell slides were also fixed by 4% PFA, permeabilized by 0.5% Triton X-100, and blocked by 1% BSA. Then, cell slides were treated with primary antibody, fluorescent secondary antibody (Runx2 and OPN, excitation = 647 nm), FITC, and DAPI in turn. Protein fluorescence images were observed by CLSM (TCS SP8, Leica, Germany). The number of parallel samples was 3.

### Statistical analysis

All of the data in this study were presented as means ± SD. All of the analyses were performed by GraphPad Prism 8 (GraphPad, USA) and analyzed by *t* test. The significant difference was considered if *P* < 0.05 (*), *P* < 0.01 (**), and *P* < 0.001 (***).

## Data Availability

Data will be made available on request.
