# Supplementary material for: Bioceramic Surface Topography Regulating Immune Osteogenesis
Source: BME Front. 2025 Jan 23;6:0089. doi: 10.34133/bmef.0089 (PMC11756600; doi:10.34133/bmef.0089)
Supplement: Supplementary 1 — Figs. S1 and S2 Table S1 [file bmef.0089.f1.docx]

SUPPLEMENTARY MATERIALS


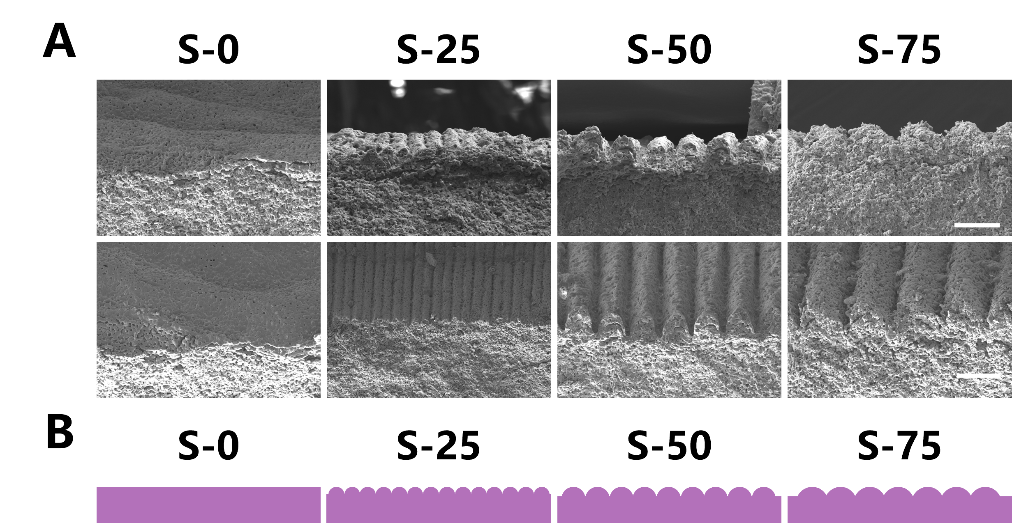


**Fig. S1. Surface morphology of bioceramics with different spaced micro-groove structures.** (A) SEM of bioceramics with different spaced micro-groove structures (Scale bar = 50 μm). (B) Schematic diagram of surface of bioceramics with different spaced micro-groove structures.


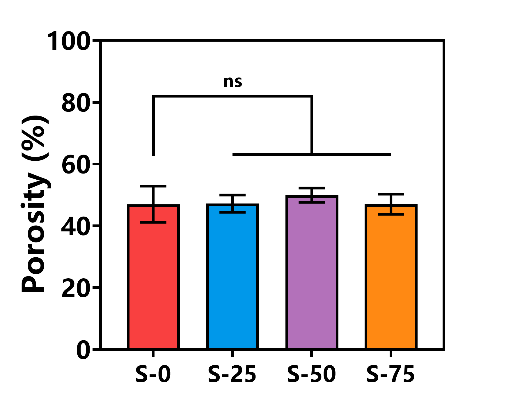


**Fig. S2. Porosity of bioceramics with different spaced micro-groove structures.** Data= means ± SD, n = 3. *p < 0.05, **p < 0.01, ***p < 0.001.

**Table. S1. Surface roughness of bioceramics with different spaced micro-groove structures.**

| Sample | Surface roughness (μm) | | |
| --- | --- | --- | --- |
| S-0 | 47.83618 | 42.48665 | 56.22152 |
| S-25 | 51.56024 | 48.26989 | 57.36434 |
| S-50 | 37.06216 | 40.83042 | 36.94257 |
| S-75 | 39.97163 | 42.49936 | 41.48432 |
